# Association Between Body Mass Index Combined with Albumin: creatinine Ratio and All-cause Mortality in Chinese Population

**DOI:** 10.1038/s41598-017-11084-5

**Published:** 2017-09-07

**Authors:** Jiachuan Xiong, Jinwei Wang, Jinghong Zhao, Luxia Zhang

**Affiliations:** 1 Renal Division, Department of Medicine, Peking University First Hospital, Beijing, China; 2 Peking University Institute of Nephrology, Peking University Health Science Center, Beijing, China; 3 Department of Nephrology, Institute of Nephrology of Chongqing and Kidney Center of the People’s Liberation Army, Xinqiao Hospital, Third Military Medical University, Chongqing, 400037 P.R. China

## Abstract

The association between body mass index (BMI) combined with albumin: creatinine ratio (ACR) and all-cause mortality in the general population has not been established. To address this, we examined a representative sample from the general population of China. The study included 46,854 participants with a follow-up of 4.6 years. Compared to the normal weight with ACR <10 mg/g group (the reference group), the crude hazard ratios (HRs) for all-cause mortality for the underweight with ACR >10 mg/g, normal weight with ACR >10 mg/g, overweight with ACR >10 mg/g, and obese with ACR >10 mg/g groups, were 2.22 (95% CI, 1.41 to 3.49), 1.70 (95% CI, 1.42 to 2.04), 1.52 (95% CI, 1.22 to 1.89), and 2.05 (95% CI, 1.45 to 2.89), respectively. After multivariable adjustments for age, race, comorbidities, and baseline eGFR, the HRs for the underweight with ACR >10 mg/g and normal weight with ACR >10 mg/g groups were 1.85 (95% CI, 1.17 to 2.91) and 1.36 (95% CI, 1.13 to 1.63), respectively. The results indicate that BMI combined with ACR can better predict all-cause mortality than BMI alone in the general Chinese population. Underweight and normal weight people with elevated ACR are at a higher risk of all-cause mortality than those in the same BMI category with ACR <10 mg/g.

## Introduction

Obesity is a medical problem in which body fat has accumulated to the extent. Commonly caused by a combination of excessive food energy intake, lack of physical activity, and genetic susceptibility^1^, it may have a negative effect on health that can reduce life expectancy and increase health problems^2^. Body mass index (BMI: the weight in kilograms divided by the square of the height in meters) is a common indicator used to define obesity. According to a World Health Organization (WHO) survey, more than 1 billion adults are overweight (BMI ≥25.0), and of these, at least 300 million are obese (BMI ≥30.0). The prevalence of obesity continues to increase rapidly in both developed and developing countries. In the United States, 34.9% of adults aged 20 years or older were obese in 2011–2012^3^. An analysis from the China National Diabetes and Metabolic Disorders Study showed that, in 2012, 31.4% and 12.2% of Chinese adults (approximately 299.5 and 116.2 million) were overweight and obese, respectively^4^. Another study showed that, worldwide, 3.4 million deaths, 4% of years of life lost, and 4% of disability-adjusted life-years could be attributed to overweight and obesity^5^. Obesity, especially central obesity, is closely associated with many metabolic disorders, such as impaired glucose tolerance, hypertension, dyslipidemia, and proinflammatory status^6^.

Many epidemiologic studies, mostly from populations of European origin, have evaluated the associations between BMI and a wide range of health outcomes. However, it has been identified that associations between BMI and health outcomes may differ between Asian and European populations^7^. Zheng *et al*.^8^ evaluated the association between BMI and risk of death in more than one million Asian individuals and concluded that underweight was associated with a substantially increased risk of death in two Asian populations. However, the limited data from the Chinese population cannot adequately explain the association between BMI and risk of cause-specific death. Moreover, there is no current large representative sample available to evaluate the association between BMI and all-cause mortality in the general Chinese population.

Albumin: creatinine ratio (ACR) is an important marker for kidney injury and a sensitive parameter to detect and identify low levels of proteinuria. ACR is also a sensitive marker for various diseases. A community-based cohort study showed that an elevated ACR could increase the risk of venous thromboembolism^9^. In people with pre-diabetes progressing to diabetes, an elevated ACR is an important adverse predictor of glycemic outcomes^10^. Urinary albumin is a marker of future blood pressure problems and hypertension in the general population, and an independent predictor of future increases in systolic blood pressure^11^. It is also an independent risk factor for 5-year mortality in chronic kidney disease (CKD)^12^. Gutiérrez reported that higher urinary albumin excretion is associated with increased risk of coronary heart disease among black adults^13^. A recent meta-analysis of 14 studies involving 105,872 participants found that an ACR ≥1 · 1 mg/mmol (10 mg/g) was an independent predictor of mortality risk in the general population^14^.

Both BMI and ACR are important predictors of mortality risk in the general population. Previous studies had showed that there was a strong association between microalbuminuria and obesity. And microalbuminuria and obesity may have additive effects on the relative risk of death^15, 16^. However, no previous study has predicted mortality risk in the general population using BMI combined with ACR. We hypothesized that BMI combined with ACR would improve the accuracy of the predictive value of death. In the current study, we aimed to evaluate the association of BMI combined with ACR to predict the risk of all-cause mortality in the Chinese population.

## Results

### Baseline Characteristics of Study Participants

Baseline characteristics of participants, categorized by BMI and stratified by ACR, are described in Table 1. The median age of the cohort was 49.7 ± 15.2 years, and more than half (57.3%) were women. The mean BMI was 23.9 ± 3.7 kg/m^2^. More than three-quarters of the study population reported that they had never smoked. More than half of the participants came from cities and towns and were educated to high school level or above. Reported hypertension and diabetes among the enrolled participants were 34.3% and 8.0%, respectively. Of those study participants with hypertension, 67% were in the highest BMI category (≥30.0 kg/m^2^), while only 13.8% were in the lowest BMI category (<18.5 kg/m^2^). As BMI increased, participants had higher systolic and diastolic blood pressures; increased uric acid, triglyceride and LDL cholesterol levels; and decreased average eGFR.

**Table 1:** Baseline Characteristics by Categories of BMI and ACR.

	Underweight ACR < 10 mg/g	Normalweight ACR < 10 mg/g	Overweight ACR < 10 mg/g	Obese ACR < 10 mg/g	Underweight ACR ≥10 mg/g	Normalweight ACR ≥10 mg/g	Overweight ACR ≥10 mg/g	Obese ACR ≥10 mg/g	Total
	(BMI < 18.5)	(BMI 18.5–24.9)	(BMI 25.0–29.9)	(BMI ≥30.0)	(BMI < 18.5)	(BMI 18.5–24.9)	(BMI 25.0–29.9)	(BMI ≥30.0)	
	N = 1639	N = 19122	N = 8596	N = 1345	N = 689	N = 8956	N = 5282	N = 1225	46854
Age(y)	42.9 ± 17.5	47.2 ± 15.1	51.0 ± 13.5	50.9 ± 14.3	48.1 ± 19.2	51.2 ± 15.8	54.7 ± 13.3	54.6 ± 13.8	49.7 ± 15.2
Men	607(37.0%)	8714(45.6%)	4328(50.3%)	607(45.1%)	207(30.0%)	3118(34.8%)	1997(37.8%)	427(34.9%)	20005(42.7%)
Rural residents	856(52.2%)	9250(48.4%)	3670(42.7%)	639(47.5%)	328(47.6%)	4088(45.6%)	2203(41.7%)	586(47.8%)	21620(46.1%)
Educated to high school or above	814(49.7%)	9113(47.7%)	4063(47.3%)	580(43.1%)	311(45.1%)	3470(38.7%)	1970(37.3%)	397(32.4%)	20718(44.2%)
Current smoker	313(19.1%)	4781(25.0%)	2369(27.6%)	342(25.4%)	106(15.4%)	1744(19.5%)	1116(21.1%)	262(21.4%)	11033(23.5%)
Hypertension	173(10.6%)	4120(21.7%)	3784(44.1%)	809(60.2%)	147(21.4%)	3045(34.1%)	3029(57.4%)	911(74.4%)	16018(34.3%)
Diabetes	40(2.4%)	838(4.4%)	682(7.9%)	142(10.6%)	34(4.9%)	760(8.5%)	760(14.4%)	212(17.3%)	3468(8.0%)
Weight (kg)	45.4 ± 5.4	57.8 ± 8.9	71.1 ± 8.5	84.6 ± 13.1	45.0 ± 5.9	57.1 ± 9.3	70.0 ± 11.5	83.1 ± 11.2	62.3 ± 12.7
Height (cm)	160.7 ± 9.2	161.5 ± 8.6	162.3 ± 8.9	161.4 ± 9.3	159.3 ± 11.7	160.1 ± 8.1	160.5 ± 8.4	160.0 ± 9.1	161.2 ± 8.7
Body-mass index (kg/m²)	17.5 ± 0.8	22.1 ± 1.8	26.9 ± 1.4	32.3 ± 3.5	17.5 ± 0.8	22.2 ± 1.8	27.0 ± 1.4	32.4 ± 3.0	23.9 ± 3.7
Systolic blood pressure (mm Hg)	112.4 ± 15.7	121.3 ± 17.6	131.9 ± 19.1	138.1 ± 20.0	119.3 ± 20.0	127.1 ± 22.0	137.6 ± 21.8	145.1 ± 22.3	127.0 ± 20.7
Diastolic blood pressure (mm Hg)	71.6 ± 9.6	77.0 ± 10.3	83.4 ± 10.7	87.7 ± 11.7	74.2 ± 11.2	79.4 ± 11.5	86.0 ± 11.7	91.2 ± 12.4	80.1 ± 11.7
Uric acid (μmol/L)	293.5 ± 81.4	297.6 ± 90.5	317.4 ± 97.1	322.8 ± 105.7	275.5 ± 83.1	281.3 ± 89.5	306.1 ± 97.9	319.7 ± 103.9	298.5 ± 93.1
Triglyceride (mmol/L)	1.0 ± 0.8	1.3 ± 1.5	1.7 ± 1.7	1.8 ± 1.3	1.1 ± 1.3	1.4 ± 1.3	1.9 ± 1.8	2.1 ± 1.8	1.5 ± 1.5
LDL cholesterol (mmol/L)	2.6 ± 0.9	2.9 ± 0.9	3.2 ± 0.9	3.3 ± 0.9	2.6 ± 0.8	2.9 ± 1.0	3.1 ± 0.9	3.2 ± 0.9	2.9 ± 1.0
HDL cholesterol (mmol/L)	1.6 ± 0.4	1.5 ± 0.4	1.3 ± 0.3	1.2 ± 0.4	1.6 ± 0.5	1.4 ± 0.4	1.3 ± 0.3	1.2 ± 0.3	1.4 ± 0.4
Creatinine (μmol/L)	70.9 ± 17.7	73.7 ± 18.5	76.4 ± 21.5	75.4 ± 16.3	74.1 ± 19.9	74.9 ± 23.8	76.4 ± 21.7	77.0 ± 20.9	74.8 ± 20.6
eGFR (mL/min per 1·73 m²)	110.2 ± 31.5	104.3 ± 27.8	99.1 ± 25.6	98.7 ± 25.4	102.1 ± 30.9	99.4 ± 27.6	95.7 ± 25.8	94.3 ± 26.0	101.2 ± 27.4

Note: BMI: body mass index; ACR: albumin: creatinine ratio; LDL: Low-density lipoprotein; HDL: high-density lipoprotein; eGFR: estimate glomerular filtration rate.

### BMI and ACR with All-Cause Mortality

**Figure 1:**
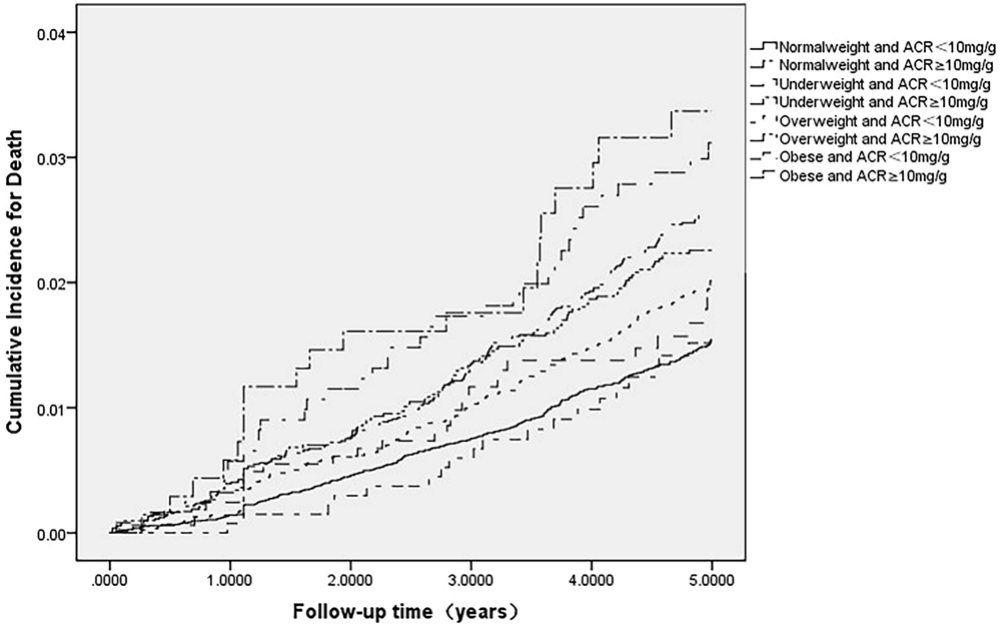
Cumulative incidence for mortality in Chinese general population. Kaplan–Meier curves for mortality for participants based on BMI and ACR stratification (Log-rank test p < 0.001).

A total of 846 deaths were reported during a 4.6-year median follow-up (mortality rate = 3.84/1000 person-years). Kaplan-Meier product-limit estimates for the cumulative incidence of death are shown in Tables 2 and 3. For the cumulative incidence of death, participants with BMI <18.5 kg/m^2^ and ACR ≥10 mg/g were among those with the highest risk, while those with BMI ≥30.0 kg/m^2^ and ACR <10 mg/g were at the lowest risk (Fig. 1 and Table 3). Table 3 also describes associations between BMI combined with ACR and mortality in the overall cohort.

**Table 2:** The association between BMI categories combined with ACR and all-cause mortality.

Variable	Total	Death	Person-years	Number of death/1000 person-years	Log-rank *P* value
Underweight and ACR < 10 mg/g	1639	28	7195	3.89	0.009
Underweight and ACR ≥10 mg/g	689	20	3068	6.52	0.020
Normalweight and ACR < 10 mg/g	19122	262	89125	2.94	0.002
Normalweight and ACR ≥10 mg/g	8956	210	41695	5.04	0.004
Overweight and ACR < 10 mg/g	8596	157	41455	3.79	0.004
Overweight and ACR ≥10 mg/g	5282	114	25342	4.50	0.006
Obese and ACR < 10 mg/g	1345	19	6515	2.92	0.007
Obese and ACR ≥10 mg/g	1225	36	5929	6.07	0.014
Total	46854	846	220324	3.84	0.002

Note: BMI: body mass index; ACR: albumin: creatinine ratio.

**Table 3:** The association of BMI and ACR categories with mortality in Chinese general population.

	Model 1		Model 2		Model 3		Model 4	
	HR (95% CI)	P value	HR (95% CI)	P value	HR (95% CI)	P value	HR (95% CI)	P value
**ACR <10** **mg/g**								
Underweight	1.34(0.91, 1.97)	0.15	1.50(1.01, 2.21)	0.04	1.49(1.00, 2.22)	0.05	1.49(1.00, 2.22)	0.05
Normalweight	Ref		Ref		Ref		Ref	
Overweight	1.29(1.06, 1.57)	0.01	1.14(0.93, 1.39)	0.21	1.08(0.89, 1.32)	0.44	1.09(0.89, 1.33)	0.42
Obese	0.99(0.62, 1.57)	0.95	0.87(0.55, 1.39)	0.56	0.81(0.51, 1.29)	0.38	0.81(0.51, 1.30)	0.38
**ACR ≥10** **mg/g**								
Underweight	2.22(1.41, 3.49)	<0.001	1.82(1.15, 2.87)	0.01	1.85(1.17, 2.91)	0.01	1.85(1.17, 2.91)	0.01
Normalweight	1.70(1.42, 2.04)	<0.001	1.39(1.16, 1.67)	<0.001	1.35(1.13, 1.63)	<0.001	1.36(1.13, 1.63)	<0.001
Overweight	1.52(1.22, 1.89)	<0.001	1.14(0.91, 1.42)	0.26	1.06(0.84, 1.32)	0.64	1.06(0.84, 1.33)	0.62
Obese	2.05(1.45, 2.91)	<0.001	1.54(1.09, 2.19)	0.02	1.36(0.95, 1.94)	0.10	1.36(0.95, 1.96)	0.09

Note: Hazard ratios (95% CIs) of all-cause mortality associated with BMI categories in crude and multivariable-adjusted Cox models: crude (model 1), age, sex (model 2); (model 3); model 2 plus hypertension and diabetes; and model 3 plus baseline eGFR adjusted (model 4).

When ACR was used as a stratifying factor, all ACR <10 mg/g groups had a linear relationship between BMI and risk of death; as the BMI increased, the risk of all-cause mortality decreased. However, in ACR ≥10 mg/g groups, the HRs of mortality tended to increase as the BMI increased. Compared to the normal weight (BMI 18.5–24.9 kg/m^2^) with ACR <10 mg/g group (the reference group), the crude HRs for all-cause mortality for the underweight (BMI <18.5 kg/m^2^) with ACR <10 mg/g, overweight (BMI, 25.0–29.9) with ACR <10 mg/g, and obese (BMI ≥30.0) with ACR <10 mg/g groups were 1.34 (95 percent confidence interval [CI], 0.91 to 1.97), 1.29 (95% CI, 1.06 to 1.57), and 0.99 (95% CI, 0.62 to 1.57), respectively. Compared to the same reference group, the crude HRs for all-cause mortality for the underweight with ACR >10 mg/g, normal weight with ACR >10 mg/g, overweight with ACR >10 mg/g, and obese with ACR >10 mg/g groups were 2.22 (95% CI, 1.41 to 3.49), 1.70 (95% CI, 1.42 to 2.04), 1.52 (95% CI, 1.22 to 1.89), and 2.05 (95% CI, 1.45 to 2.89), respectively.

Multivariable-adjusted Cox models were used in our analysis. After multivariable adjustments for age, race, comorbidities, and baseline eGFR, the underweight with ACR >10 mg/g and normal weight with ACR >10 mg/g groups still showed a strong association with all-cause mortality, with HRs of 1.85 (95% CI, 1.17 to 2.91) and 1.36 (95% CI, 1.13 to 1.63), respectively. The lowest adjusted mortality (model 5) was observed in the obese with ACR <10 mg/g group where the HR was 0.81 (95% CI, 0.51 to 1.30). The highest adjusted mortality (model 4) was observed in the BMI <18.5 kg/m^2^ with ACR ≥10 mg/g group where the HR was 1.85 (95% CI, 1.17 to 2.91). Analysis of the interaction between BMI and ACR was also performed but a significant difference was not observed.

## Discussion

In the analysis of 46,854 participants, we found that BMI combined with ACR was strongly associated with all-cause mortality in the general population. People with a low BMI and ACR ≥10 mg/g were among those at highest risk. For cumulative incidence of death, the BMI ≥30.0 kg/m^2^ with ACR ≥10 mg/g group was at the lowest risk, the BMI 18.5–24.9 kg/m^2^ with ACR ≥10 mg/g group was among those at the highest risk, and the BMI ≥30.0 kg/m^2^ with ACR <10 mg/g group was at the lowest risk.

In the general population, overweight and obesity can cause and accelerate the progression of hypertension^17^, diabetes^18^, cardiovascular disease^19^, CKD^20^, other comorbidities, and even death. The main pathophysiological characteristics of obese people are that blood volume and cardiac output are increased, which can result in eccentric left ventricular (LV) hypertrophy^21^. Visceral obesity is strongly correlated with insulin resistance because visceral obesity can disrupt the balance between the secretion of pro- and anti-atherogenic substances. As a result, the production of pro-atherogenic substances and inflammatory cytokines is markedly increased, interfering with insulin signaling, and contributing to the development of insulin resistance^22^. Thus, people with some or all of the metabolic syndrome components have an increased risk of developing type 2 diabetes mellitus^23^. Overweight and obesity can also have an important impact on the kidneys via hemodynamic and hormonal effects. This can lead to glomerular hyperperfusion (increased renal plasma flow) and glomerular hyperfiltration (increased GFR)^24^, renal hemodynamic changes that contribute to the development of obesity-related glomerulopathy^25^. Conditions coexisting with obesity, such as diabetes and hypertension, can also promote the development of CKD^26^.

A large number of studies have evaluated the relationship between overweight and obesity and mortality for all-causes^8, 27^ or special diseases^28^, but the results are inconsistent. Some studies found that obesity can increase the risk of mortality, while others reported that being overweight (BMI 25–29.9 kg/m^2^) or having class I obesity (BMI 30–34.9 kg/m^2^) were associated with lower mortality. BMI is not a perfect measure of body fat and does not distinguish well between people with abdominal obesity, athletes, and pregnant women^29^. However, it has been commonly used for years in clinical practice and epidemiologic research worldwide as an indicator of obesity, due to its simplicity and ease of calculation. Pooled analysis of several studies reported that in comparison to normal weight category, being overweight (BMI 25–29.9 kg/m^2^) or with class I obesity (BMI 30.0–34.9 kg/m^2^) were not associated with higher mortality^30^. Overall, our results relating to BMI were similar to previous studies. Differences could be attributed to the shorter follow-up time available, and the different ethnic population studied.

ACR is a key marker for monitoring kidney function when a kidney is dysfunctional. An injured filtration barrier can result in proteinuria. Hypertension and diabetes (diabetes kidney disease) may cause albuminuria. ACR has been widely identified as an independent risk factor for kidney dysfunction. Recent studies have determined that increased ACR can contribute to a higher risk of cardiovascular and all-cause mortality. Konta *et al*.^31^ conducted a community-based longitudinal study in the Japanese population, with 3,445 participants (mean age 62.6 years) and a 7-year follow-up. They found that an ACR ≥30 mg/g increased the all-cause and cardiovascular mortality 1.69-fold and 2.27-fold, respectively. Chen *et al*.^32^ investigated the relationship between albuminuria and mortality in a middle-aged-to-elderly Chinese population and found that relative risks (RR) of cardiovascular mortality were 2.72 in the microalbuminuria group, and 4.87 in the macroalbuminuria group. Adjusted RRs for all-cause mortality for the two groups were 2.01 and 3.76, respectively.

Increased ACR is also associated with the metabolic syndrome. In a study of non-diabetic black African individuals, Okpechi *et al*.^33^ reported a significant increase in ACR with increasing numbers of metabolic syndrome traits, with ACR increasing four-fold in those with four or more traits compared with participants with no metabolic syndrome traits. Premicroalbuminuria is also a metabolic risk marker in women with polycystic ovary syndrome. Women with polycystic ovary syndrome who had an ACR >7 mg/g had significantly higher blood pressure and alanine aminotransferase levels than those with an ACR ≤7 mg/g^34^. Microalbuminuria reflects generalized endothelial dysfunction and evidence suggests that an elevated ACR is a critical biomarker for various diseases. Velde *et al*.^35^ even suggested that albuminuria should be added to the present criteria defining the metabolic syndrome in the PREVEND study.

Obesity was related to an increased albumin excretion rate^36^. Previous studies suggest that abdominal obesity may independently associate with microalbuminuria^37, 38^.

Both BMI and ACR are important factors in predicting all-cause mortality. However, there is a lack of data to investigate the relationship between these two parameters together and all-cause mortality. Our hypothesis was that the use of ACR stratification would increase the accuracy of BMI for predicting death. We used a cohort study to determine the association between mortality and combined BMI and ACR categories in the general Chinese population. A previous study found that the average BMI of the general Chinese population was lower than that of European and American-African populations. Underweight may be caused by malnutrition and chronic wasting diseases that can increase the risk of death. In a longevity study, compared with persons with normal BMI, persons whose BMI were below 18.5 kg/m^2^ was at increased risk of death over 30 years^39^. Some mechanisms may explain why underweight patients have higher mortality. First, underweight patients have decreased physiologic reserve, which may lower their ability to withstand insults, thus make them more vulnerable to adverse events^40^. More hypertension or diabetes may be diagnosed in people with an ACR ≥10 mg/g, and these diseases may also increase the risk of death. In addition, Studies reported underweight patients were with an increased risk of CVD or cancer mortality^41^. At last, a large part of underweight patients was diagnosed malnutrition that also can increase mortality risk^42^.

The risk of death for overweight and obese people with an ACR ≥10 mg/g is higher than for overweight or obese people with an ACR <10 mg/g, but not higher than for underweight people (BMI <18.5 kg/m^2^) with an ACR <10 mg/g. This indicates that the BMI alone did not increase the risk of all-cause mortality, as the risk was altered when ACR was used to stratify the population and when multivariable adjustments were made for age, race, comorbidities, and baseline eGFR. Thus, BMI combined with ACR can increase the accuracy of predicting the risk of death. Previous studies have showed obesity or microalbuminuria alone is a predictor of death. Our study demonstrated that obesity people with ACR under ACR <10 mg/g may not have a high risk of death, but those with ACR over ACR <10 mg/g may have. This indicate that overweigh people may benefit from the weight that can withstand adverse events, but when the microalbuminuria is shown and ACR over 10 mg/g, those people will be at a high risk of all-cause mortality, and the same results in underweight patients. Our study firstly combined the BMI and ACR parameter to predict the risk of death in general population. Through the stratification according to weight and ACR, we may know that those people whether they are at a low or a high risk of death, this is the most different from other studies. In another word, using both BMI and ACR, we can predict the risk more accurately than BMI or ACR alone.

However, there are some limitations that need to be acknowledged. The average follow-up time of our study was only 4.6 years, which was shorter than other similar studies, and may have had an impact on the findings. Our study also cannot explain the long-term effect of BMI on mortality, as some of the ill-effects of excess adipose tissue take many years to manifest. In addition, we did not examine cause-specific mortality, which could have provided information on the different effects of low and high BMI on a variety of outcomes. Not excluding participants with diagnosed diseases at baseline may have increased the association between BMI combined with ACR and mortality.

In conclusion, BMI combined with ACR can better predict all-cause mortality than BMI alone in the general Chinese population. Underweight and normal-weight people with elevated ACR are at a higher risk of all-cause mortality than those in the same BMI category with ACR <10 mg/g. Long-term follow-up studies are needed to confirm our results.

## Subjects and Methods

### Study Population

Participants came from a cross-sectional survey of a nationally representative sample of Chinese adults. The survey used a multistage, stratified sampling method to obtain a representative sample of people aged 18 years or older in the general population. The survey was undertaken from January 2007 to October 2010. A total of 50,550 people was invited to participate, and 47,204 completed the survey and examination. Participants who had missing information on height or weight or insufficient sample collection were excluded from this study. The remaining 46,854 participants were included in the final analysis and followed up in our cohort.

### Baseline Examination and Definitions

Sociodemographic data (such as age, sex, income, and education level) and the presence of comorbidities were obtained from the questionnaire. Anthropometric measurements and blood pressure were taken by trained staff who were taught the methods and processes of the study. Biological specimens were collected and examined for indicators of kidney damage and possible risk factors. All blood and urine samples were analyzed at the central laboratory in each province. All the study laboratories had successfully completed a standardization and certification program. Urinary albumin and creatinine were measured from a fresh morning spot urine sample or morning urine sample stored at 4 °C for less than one week. Albuminuria was measured with immunoturbidimetric tests. Urinary creatinine was measured with Jaffe’s kinetic method. The urinary albumin to creatinine ratio (ACR; mg/g creatinine) was calculated. The eGFR was calculated with an equation developed by adaptation of the Modification of Diet in Renal Disease (MDRD) equation based on data from Chinese chronic kidney disease patients. Serum total cholesterol, LDL cholesterol, HDL cholesterol, triglycerides, and uric acid were measured with commercially available reagents. The laboratories used a timed-endpoint colorimetric method to measure LDL cholesterol and HDL cholesterol. More details of the methods used were described previously^43^. Weight and height measurements obtained on cohort participants were used to calculate BMI as the weight in kilograms divided by the height in meters squared.

### Outcomes

Our study obtained informed consent from individual participants to use their social identification number, first name, last name, and birth date to enable potential linkages for future research studies. All study participants were followed up from the screening date until December 31, 2013 or their date of death. When a death was recorded during follow-up, between January 1, 2008 and December 31, 2013, the cause of death certification was obtained from the Chinese Center for Disease Control and Prevention Cause of Death Reporting System^44^. We use the social identification number to match the variable number, and information such as name, gender, age, and address to check the accuracy of the matching.

### Ethical Statement

The ethics committee of the Peking University First Hospital approved the study. All participants gave written informed consent prior to data collection. This study was performed according to the guidelines of Peking University First Hospital, which abides by the Helsinki Declaration on ethical principles for medical research involving human participants.

### Statistical Analysis

Descriptive analyses were performed using means ± SDs, medians (interquartile ranges), and proportions, as appropriate. Cumulative incidence curves for risk of death were plotted using the Kaplan-Meier method, with differences across BMI categories assessed using the log-rank test. The association between combined BMI with ACR and the risk of death was analyzed with the Cox proportional-hazards regression model, with a categorical representation of BMI as the predictor variable. BMI groups for the analysis were defined as underweight (BMI < 18.5 kg/m^2^), normal weight (BMI 18.5–24.9 kg/m^2^), overweight (BMI 25.0–29.9 kg/m^2^) and obese (BMI ≥ 30.0 kg/m^2^) as advocated by the WHO. ACR ≥10 mg/g and ACR <10 mg/g categories were used to stratify the groups further. Group 1: BMI <18.5 kg/m^2^ and ACR <10 mg/g, Group 2: BMI <18.5 kg/m^2^ and ACR ≥10 mg/g, Group 3: BMI 18.5–24.9 kg/m^2^ and ACR <10 mg/g, Group 4: BMI 18.5–24.9 kg/m^2^ and ACR ≥10 mg/g, Group 5: BMI 25.0–29.9 kg/m^2^ and ACR <10 mg/g, Group 6: BMI 25.0–29.9 kg/m^2^ and ACR ≥10 mg/g, Group 7: BMI ≥30.0 kg/m^2^ and ACR <10 mg/g, Group 8: BMI ≥30.0 kg/m^2^ and ACR ≥10 mg/g. The BMI range of 18.5 to 24.9 kg/m^2^ and ACR <10 mg/g (Group 3) was used as the reference group when we estimated hazard ratios (HRs) and 95% confidence intervals for groups. We also adjusted for potential confounders including baseline age, sex, educational level, urban or rural residence, and smoking status. The effect of potential confounders was analyzed by constructing models with incremental adjustments: unadjusted (model 1); age, sex (model 2); model 2 plus hypertension and diabetes (model 3); and model 3 plus baseline eGFR adjusted (model 4).

Statistical analyses were performed using Statistical Product and Service Solutions (SPSS) version 20 (SPSS Inc, Chicago, IL).
